# An Artificial Nose

**Published:** 1894-10

**Authors:** P. W. Moriarity

**Affiliations:** Boston, Mass.


### International Dental Journal.

Vol. XV. October, 1894. No. 10.

### Original Communications.[1]

### AN ARTIFICIAL NOSE.[2]

BY P. W. MORIARTY, D.M.D., BOSTON, MASS.

On January 10, 1894, J. W. T., aged twenty-three, the patient, whom I present this evening, came to the infirmary of the Harvard Dental School to have an obturator made to relieve a deformity due to disease which first made its appearance eight years ago.

A plate made with several teeth attached restored the voice and covered the perforation in the hard palate.

By glancing at the young man's face you can see the distressing deformity due to this disease (Fig. 1).

To hide this I urged upon him the advisability of having an artificial nose made.

You are familiar with the efforts of Drs. Brackett, Kingsley, and others in this direction.

An impression of the deformed nose and adjacent parts was taken in plaster and a cast made.

A wax nose was made for a model, and by additions and carvings and frequent trials upon the patient it was brought to the shape desired.

[1] The editor and publishers are not responsible for the views of authors of papers published in this department, nor for any claim to novelty, or otherwise, that may be made by them. No papers will be received for this department that have appeared in any other journal published in the country.

[2] Exhibited before the Harvard Odontological Society April 26, 1894.

A metal die and counter-die of the prepared wax nose was made, and a thin platinum shell struck up. Nostrils were soldered to it. This was covered with continuous gum body and baked.

The flesh-colored enamel was then added and the piece again baked.

Hydrofluoric acid was used to soften the color and remove the glaze.

Owing to the small size of the nasal orifices it was impossible to attach the nose to the obturator, or by springs inserted through the nostrils.

A rim of German silver was made shaped to the upper edge of the artificial nose, and extending about three-quarters of an inch downward on each side. To this a pair of spectacles was soldered, and the rim fastened with small rivets to the platinum shell, the spectacles with an elastic band passing around the head and holding the nose in position (Fig. 2).

Mr. A. G. Smith, one of our students, had charge of the case. That you may judge as to the success of the appliance, the patient has kindly consented to appear before you this evening.

